# Ancient Reef Traits, a database of trait information for reef-building organisms over the Phanerozoic

**DOI:** 10.1038/s41597-022-01486-0

**Published:** 2022-07-20

**Authors:** Nussaïbah B. Raja, Danijela Dimitrijević, Mihaela Cristina Krause, Wolfgang Kiessling

**Affiliations:** GeoZentrum Nordbayern, Department of Geography and Geosciences, Friedrich-Alexander-Universität Erlangen-Nürnberg, Erlangen, Germany

**Keywords:** Palaeoecology, Biodiversity

## Abstract

Trait-based approaches are increasingly relevant to understand ecological and evolutionary patterns. A comprehensive trait database for extant reef corals is already available and widely used to reveal vulnerabilities to environmental disturbances including climate change. However, the lack of similar trait compilations for extinct reef builders prevents the derivation of generalities from the fossil record and to address similar questions. Here we present the Ancient Reef Traits Database (ARTD), which aims to compile trait information of various reef-building organisms in one single repository. ARTD contains specimen-level data from both published and unpublished resources. In this first version, we release 15 traits for 505 genera and 1129 species, comprising a dataset of 17,841 trait values of Triassic to mid-Holocene scleractinian corals, the dominant reef-builders in the modern ocean. Other trait data, including for other reef-building organisms, are currently being collated.

## Abstract (summary)

**Table:** 

Measurement(s)	fossil coral traits
Technology Type(s)	digital curation
Sample Characteristic - Organism	Scleractinian corals
Sample Characteristic - Location	Global

## Background & Summary

Trait-based approaches are one way to correlate different characteristics, or traits, of organisms to environmental changes with the ultimate goal to predict community and whole-ecosystem responses to such changes. Traits of reef corals have been explored to identify their life-history strategies^1^ and extinction risk^2,3^. Several palaeontological studies have examined trait-based extinction using the fossil record^4–6^. Changes in ancient reef ecosystems can provide valuable insights into drivers of reef proliferation and decline. Understanding why fossil reefs collapsed and how they recovered may help to inform conservation activities in modern coral reefs in light of anthropogenic climate change. Many reefs of the Phanerozoic eon (the last 540 million years) can be considered analogues of modern tropical coral reefs with regards to reef architecture and environmental controls^7^. Phanerozoic reef-building is not restricted to scleractinian corals but also a multitude of other sessile hypercalcifying animals (animals with a large skeletal to biomass ratio) such as extinct coral clades, calcifying sponges and rudist bivalves.

In the first release of this database, we focus on scleractinian corals, which have a rich fossil record from the Middle Triassic (~245 Ma) and became the dominant reef builders in the Late Triassic (~225 million years ago)^8^. Scleractinian corals have an extensive fossil record due to their calcified skeletons. Accordingly, morphological traits are well preserved and several of those traits have been shown to be linked to species extinction risk: examples are corallite integration, corallite diameter, growth rate (linked to morphology or measured directly from growth bands), and colony longevity (linked to size)^3,9–12^. Spatial and environmental traits are also important predictors of extinction risk: habitat breadth and maximum water depth are two key parameters linked to climate change vulnerability in corals^9^.

The traits of extant reef corals have been compiled in the openly accessible Coral Traits Database (CTD)^13,14^, which in addition to morphological traits, also contains data on physiology, biology and reproduction. However, there is no single, exhaustive resource for fossil reef building organisms including extinct scleractinian corals and the traits of extinct corals are currently scattered in online repositories and research publications. Some trait compilations that cover only one type of trait (i.e., corallite integration) and/or span one period (i.e., Triassic) have previously been published^15^ but are not openly available. The online resource Corallosphere (www.corallosphere.org) contains a description of genus-specific traits for both extinct and extant scleractinian corals, while the Paleobiology Database (PBDB; www.paleobiodb.org) contains information for a limited number of traits such as inferred symbiotic status or preferred environment. The database on Neogene Marine Biota of Tropical America (NMITA) mostly contains trait information of Pleistocene to Holocene scleractinian corals from the Caribbean region^16,17^. Therefore, there is a need for an updated, comprehensive and openly accessible compilation of fossil coral traits.

Here, we present the Ancient Reef Traits Database (ARTD) which fills this gap. ARTD is a unique, specimen-based compilation of reef builders’ traits. At the initial stage, the database contains 15 traits of scleractinian corals and covers the time period from the mid-Triassic until the mid-Holocene. The value of ARTD lies not just in its data coverage but in its interlinkage with other databases. ARTD is compatible with CTD and is also designed in a way that provides easy integration with the biggest resource of fossil occurrence data, namely the PBDB.

## Methods

**Fig. 1:**
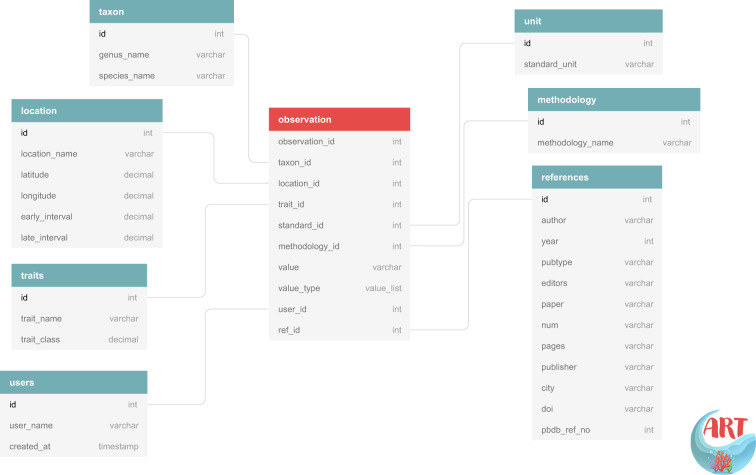
Overview of the ARTD back-end structure. The observation table is highlighted in red. The remaining tables contain supplementary information that complement the observation records.

ARTD has a similar structure as the CTD and is designed to contain specimen-level traits for identified species and genera (Fig. 1). The basic unit of entry is that of the trait of a single specimen, e.g. a coral specimen, that is also accompanied by contextual characteristics such as the geological stage(s), the present-day geographic region and the present-day coordinates of the locality in decimal degrees, in which the specimen was found. Reconstructed palaeo-coordinates were computed from the present-day coordinates using the rotation file supplied by C. Scotese in the ‘PALEOMAP PaleoAtlas for GPlates’ package^18^. These metadata are crucial for analyses of traits in a spatial, temporal and environmental context. For example, the corallite diameter or colony size of a species may vary due to genealogical trends or environmental factors^19^. A specimen can be linked to a number of observed traits as per the source of the data. In the case where there are multiple observations for a specimen or group of specimens (usually reported as such in the primary source), text-based information is entered and separated by “-“, e.g. trabecular-substyliform for columella structure. We also employ a hierarchical taxonomic structure where any inheritable trait (i.e. symbiotic status or corallite integration) of a taxon, is automatically applied to all the lower taxonomic levels and specimens of that particular taxon. For example, if a genus’ corallite integration is qualified as cerioid all its containing species and specimens are also assigned to a cerioid trait, unless exceptions are known. In the case of growth forms, heritability was assigned only to genera where one growth form has been assigned, e.g. *Isastraea* (massive) as growth forms tend to vary among some groups due to factors such as environment^20^.

The taxonomic and contextual information are stored separately and linked to the “observation” table where each entry is provided with a unique identification number (id; Fig. 1). This observation table contains the respective specimen-level traits which contains information of the trait(s) being entered, the value (numeric or character depending on the trait being entered), the value type (whether raw, aggregated, or based on the opinion of experts), and the method used to obtain the measurements (e.g. observations made in the field or laboratory). This structure is used so that multiple specimens from one specific site and one taxon can be linked several times to the same traits. For example, published articles provide several measurements of corallite widths^21^. In the case when a range of values is provided for a measured trait, the mean value is entered, except for the traits “corallite width minimum” and “corallite width maximum” where the minimum and maximum values are entered, respectively. Traits are divided using the categories that are comparable to the CTD: morphological, reproductive, or physiological. However, it is clear that only morphological traits are directly accessible in fossils, whereas the latter categories need to be inferred. Currently the only inferred trait in our database is that of symbiotic status (trait_name: “Zooxanthellate”) which relies on morphological criteria of extinct coral species as defined by Kiessling and Kocsis^22^. The original source of the entered data is also included in the database (called the primary source), and if available, the reference number for this primary source in the PBDB which can then be used to integrate both databases. The taxonomic information is automatically validated against the PBDB before each database release to ensure that the most up-to-date information is available.

## Data Records

**Fig. 2:**
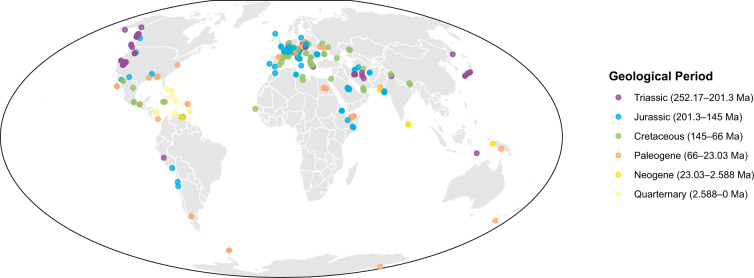
Map of localities, categorised by geological period, from which trait data of scleractinian corals were compiled in ARTD.

**Fig. 3:**
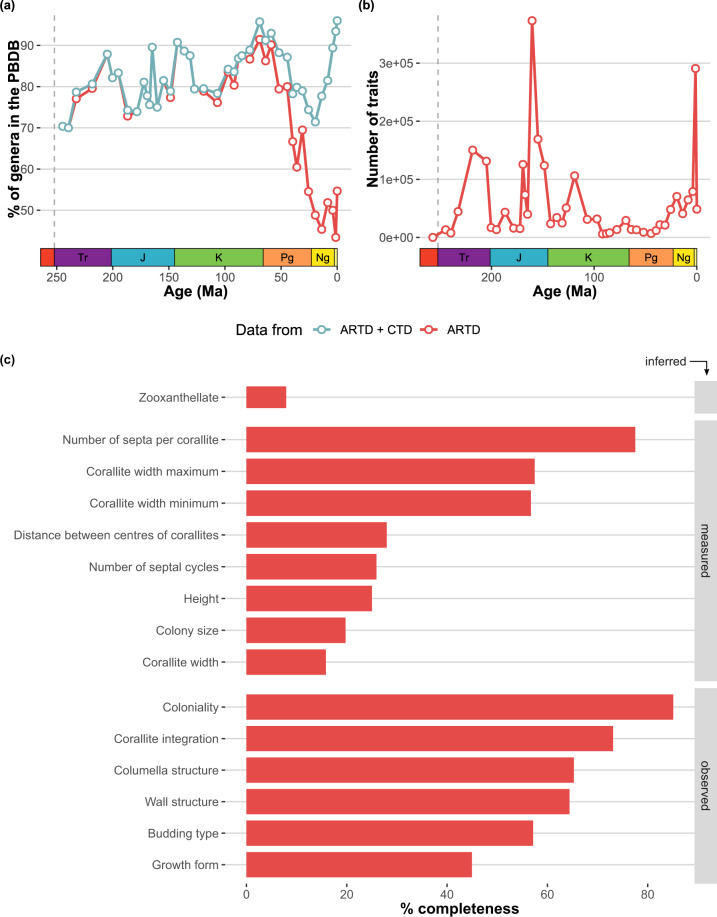
Data coverage in ARTD: (**a**) Taxonomic coverage of ART-only data and ART data combined with CTD compared to taxonomic occurrences in the PBDB; (**b**) Number of trait values in ARTD for coral genera in the PBDB; and (**c**) Data completeness of traits as a percentage of specimens in ARTD.

Here, we provide a release of 15 traits of 505 genera and 1129 species, comprising a dataset of 17,841 trait values obtained from localities around the world (Fig. 2). This first release has broad taxonomic and temporal coverage and comprises more than 70% of all coral genera in the PBDB in each geological stage since the mid-Triassic (Figs. 2, 3a). The availability of traits per specimen or taxon also varies (Fig. 3b) depending for example on the preservation of the specimen being reported in the primary source, with some traits such as corallite diameter or corallite integration more readily available than others such as colony size (Fig. 3c).

A static release of the database is available directly from the ART platform (https://art.nat.fau.de) as well as Zenodo^23^. The data release is in the form of a compressed folder containing two files:

1. **data.csv**: A csv-formatted file containing the contextual information and measurement of specimens.
2. **references.csv**: A csv-formatted file containing the bibliographic information of data sources^24–114^.

The details and descriptions of the available trait variables are available in Table 1. Up-to-date data are directly available from the database. However, as data entry and validation (see Technical Validation) is ongoing, users are recommended to use the data made available through the static releases to maximise reproducibility of analyses and results. Both the static releases^23^ and direct downloads are accompanied by the primary sources^24–114^ which should be credited.

**Table 1:** Overview of traits available in ART v1.0, including descriptions and standard categories used.

Trait class	Trait name	Inherited trait?	Description	Categories	Category descriptions	Number of observations
Morphological	Coloniality	yes	Whether mature individuals of a species form colonies or are solitary	Colonial Solitary	Mature individuals are colonial Mature individuals are solitary	1861
Morphological	Number of septa per corallite	no	The mean number of septa observed in one corallite	N/A	N/A	1747
Morphological	Corallite integration (Colony form in CTD)	yes	The general arrangement of corallites in a colony	Thamnasteroid Plocoid Subplocoid Cerioid Meandroid Flabelloid Phaceloid Dendroid Solitary	Corallites with confluent septa and lacking defined boundaries Corallites separated by coenosteum Corallites sometimes separated by coenosteum Corallites juxtaposed Corallites arranged in multiple series Corallites arranged in single series Corallites separated and subparallel Corallites separated and irregularly branching Corallum formed by only one individual	1390
Morphological	Corallite width maximum	no	Maximum diameter of the corallite	N/A	N/A	1378
Morphological	Corallite width minimum	no	Minimum diameter of the corallite	N/A	N/A	1358
Morphological	Columella structure	yes	The overall form of the central axial structure within a corallite	Spongy Trabecular Papillose Fascicular Styliform Lamellar Absent	A fine porous mass An irregular group of twisted elements, also referred to as parietal A group of rods A set of twisted lamellae A simple rod In the shape of a single lamella No columella	1088
Morphological	Wall structure	yes	The structure of skeleton enclosing a corallite	Epithecal Parathecal Septothecal Septoparathecal Synapticulothecal Absent	Corallite wall is formed by the epitheca Corallite wall formed by dissepiments Corallite wall formed by thickening of septa Corallite wall formed by thickening of septa and dissepiments Corallite wall formed by rings of synapticulae (horizontal rods between septa) No wall	972
Morphological	Growth form	yes	The shape in which the coral specimen grows	Massive Branching Platy Columnar Discoid Flabellate Fungiform Reptoid Cylindrical Turbinate Patellate Trochoid Cupolate Ceratoid Cuneiform Encrusting	Mound-shaped and hemispherical colony Colony composed of elongate projections Flattened colony with calices on only one side Pillar or finger-like colonies that do not have the secondary branches Nearly all in a single plane, horizontal wall and flat or slightly concave or convex oral surface; solitary Fan-shaped: both solitary and colonial Mushroom shaped; colonial Corallites separated by void space Creeping over some substrate, encrusting; colonial Nearly straight and of uniform diameter except in the apical region; solitary Like trochoid but with wider apical angle, about 70 degrees; solitary With still wider apical angle, about 120 degrees; broadly flattened conical in form; solitary The angle is about 40 degrees; solitary Flat base and highly convex oral surface; solitary Very slenderly conical, horn-shaped, the angle is only about 20 degrees; solitary Wedge-shaped; solitary Encrusting colony	916
Morphological	Distance between centres of corallites	no	The measured distance between the centres of two corallites	N/A	N/A	909
Morphological	Number of septal cycles	no	Number of cycles or orders in the mature corallite	N/A	N/A	647
Morphological	Height	no	The overall height of the specimen, usually a solitary coral	N/A	N/A	592
Morphological	Colony size	no	The maximum diameter of a colony	N/A	N/A	559
Morphological	Corallite width	no	Diameter of the corallite	N/A	N/A	428
Physiological	Zooxanthellate	yes	Whether the species is zooxanthellate (i.e., contains photosymbiotic zooxanthellae) or not Note: This is not directly observable and is inferred.	Zooxanthellate Azooxanthellate Apozooxanthellate	Contain zooxanthellae within their tissues Don't contain zooxanthellae within their tissues Sometimes contain zooxanthellae within their tissues	387
Reproductive	Budding type	yes	The position of new buds relative to the parent corallite wall Note: This is a morphological character that is directly observable	Intracalicular Extracalicular Both None	Occurring within the tentacle ring of the parent polyp Occurring outside the tentacle ring, with daughter corallites forming on the side of the parent corallite Both intra- and extracalicular No budding occurring	201

All data in the ARTD and included in this release are linked with published (e.g. peer-reviewed papers, taxonomic monographs, books) references^24–114^. The final dataset consists of one row per trait for each specimen entered. Each specimen is given a unique identification number (**observation_id**) which can be associated with various traits. The geographic data is available for each specimen, such as the country or region in which it was found (**location_name)**, present-day coordinates (**longitude, latitude**), reconstructed palaeo-coordinates (**paleolng**, **paleolat**), and taxonomic information (**identified_name:** as entered in the database, **accepted_name**: based on updated taxonomic information, **genus_name:** genus of the specimen as per the updated taxonomic information, **species_name**: the species name as per the updated taxonomic information). The time period identified for the specimen (**early_interval**: the first interval in which the specimen was found**, late_interval**: the last interval in which it was found**, min**: the minimum identified age**, max**: the maximum identified age) is also provided. For each specimen, the available traits are entered. Each trait (**trait_name**) is assigned to a category (**trait_class**), and a trait entry for a specimen contains the trait **value** and unit (**standard_unit**) if applicable. Additional information **(value_type)** about any measurements such as whether the entered measurement is raw or an aggregated value (mean, minimum, maximum) based on expert opinion, model-derived, the unit for the measurements (**standard_unit**), and the methodology used to obtain the data (**methodology_name)** is also provided if available. Each entry contains the identification number of the data source (**reference_no**), whose bibliographic details are provided in the **reference.csv** table, which can then be used for citations purposes.

## Technical Validation

The database is curated by the managerial board who undertake the tasks below. Curation and quality control of the data include:

### Updating taxonomic information

All taxonomic data are cross validated taxonomic names against the PBDB to ensure that reliable taxonomic data is available.

### Dealing with duplicates

All duplicated records identified based on the combination of data source, taxonomic information, location, time period, and measurement values are flagged and then removed.

### Applying inherited traits

Any newly entered traits that are identified as inherited traits are applied to all species of the genus that contains that particular trait.

### Standardising text-based fields

Text-based fields such as corallite integration or growth form are standardised (Table 1) to facilitate analysis on these traits.

### Contributor approval

Anyone wishing to contribute to the database should become a formal contributor and any observations entered by them will be associated with their user account.

The database is hosted on the server of the Friedrich-Alexander-Universität Erlangen-Nürnberg (FAU) and will be maintained on the long-term by WK, MCK and other staff members of the FAU.

## Usage Notes

**Fig. 4:**
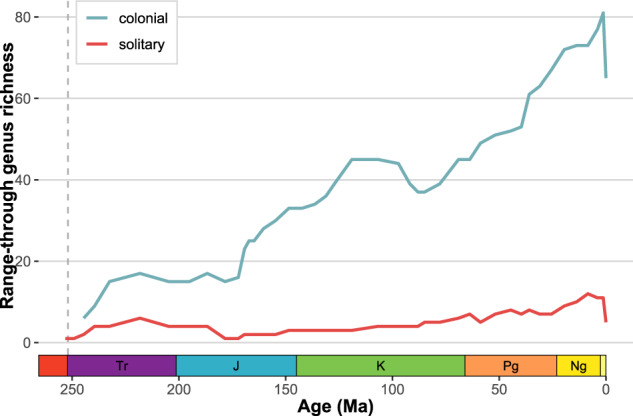
Range-through diversity of solitary and colonial corals since the Triassic using data from ARTD and the PBDB. Recent corals were not included in the range-through analysis.

ARTD offers many new opportunities for incorporating trait-based approaches in addressing macroevolutionary and macroecological questions using the fossil record of reef-building organisms. The correlation of certain traits with environmental conditions and their vulnerability or resilience to environmental change can be widely used to understand the evolution of these organisms and the reefs they build over time, their extinction risk in light of global warming and recovery after such an event. A trait-based framework allows the analysis of variation and evolution of traits within and across reef-building organisms over time and their responses to environmental change in the past. For example, the data from ARTD combined with the PBDB show that the diversity trajectories for colonial and solitary scleractinian corals were different (Spearman’s ρ = 0.241, p = 0.129 of first differences), with colonial corals showing an increase in diversity during the late Jurassic and Cretaceous but declining again in the Late Cretaceous (Fig. 4). On the other hand, the diversity of solitary corals remained relatively low compared to colonial corals.

Trait-based frameworks are also widely used in modern ecology to study the climate impacts of organisms and their extinction risk^1,14,115–118^. Focusing on such frameworks would therefore allow the integration of palaeontological and neontological data using similar concepts and methods to address urgent questions on biodiversity and extinction at multiple scales^3,119,120^. Such an integrated approach might contribute to the conservation of modern coral reefs, which are the most threatened ecosystems from climate change^121^.

## Data Availability

All the code used to generate the figures in this manuscript is available on the following GitHub repository: https://github.com/nussaibahrs/ARTD.
