# Author Correction: Ancient Reef Traits, a database of trait information for reef-building organisms over the Phanerozoic

**DOI:** 10.1038/s41597-022-01646-2

**Published:** 2022-09-02

**Authors:** Nussaïbah B. Raja, Danijela Dimitrijević, Mihaela Cristina Krause, Wolfgang Kiessling

**Affiliations:** GeoZentrum Nordbayern, Department of Geography and Geosciences, Friedrich-Alexander-Universität Erlangen-Nürnberg, Erlangen, Germany

**Keywords:** Palaeoecology, Biodiversity

Correction to: *Scientific Data*, 10.1038/s41597-022-01486-0, published online 20 July 2022.

An incorrect version of Table 1 was included in the original version of the paper, omitting some cells and disrupting the formatting and alignment of the rest of the table. The correct version of the table is shown below, which has replaced the incorrect version in the pdf and HTML versions of the article.

**Table 1:** Overview of traits available in ART v1.0, including descriptions and standard categories used.

Trait class	Trait name	Inherited trait?	Description	Categories	Category descriptions	Number of observations
Morphological	Coloniality	yes	Whether mature individuals of a species form colonies or are solitary	Colonial Solitary	Mature individuals are colonial Mature individuals are solitary	1861
Morphological	Number of septa per corallite	no	The mean number of septa observed in one corallite	N/A	N/A	1747
Morphological	Corallite integration (Colony form in CTD)	yes	The general arrangement of corallites in a colony	Thamnasteroid Plocoid Subplocoid Cerioid Meandroid Flabelloid Phaceloid Dendroid Solitary	Corallites with confluent septa and lacking defined boundaries Corallites separated by coenosteum Corallites sometimes separated by coenosteum Corallites juxtaposed Corallites arranged in multiple series Corallites arranged in single series Corallites separated and subparallel Corallites separated and irregularly branching Corallum formed by only one individual	1390
Morphological	Corallite width maximum	no	Maximum diameter of the corallite	N/A	N/A	1378
Morphological	Corallite width minimum	no	Minimum diameter of the corallite	N/A	N/A	1358
Morphological	Columella structure	yes	The overall form of the central axial structure within a corallite	Spongy Trabecular Papillose Fascicular Styliform Lamellar Absent	A fine porous mass An irregular group of twisted elements, also referred to as parietal A group of rods A set of twisted lamellae A simple rod In the shape of a single lamella No columella	1088
Morphological	Wall structure	yes	The structure of skeleton enclosing a corallite	Epithecal Parathecal Septothecal Septoparathecal Synapticulothecal Absent	Corallite wall is formed by the epitheca Corallite wall formed by dissepiments Corallite wall formed by thickening of septa Corallite wall formed by thickening of septa and dissepiments Corallite wall formed by rings of synapticulae (horizontal rods between septa) No wall	972
Morphological	Growth form	yes	The shape in which the coral specimen grows	Massive Branching Platy Columnar Discoid Flabellate Fungiform Reptoid Cylindrical Turbinate Patellate Trochoid Cupolate Ceratoid Cuneiform Encrusting	Mound-shaped and hemispherical colony Colony composed of elongate projections Flattened colony with calices on only one side Pillar or finger-like colonies that do not have the secondary branches Nearly all in a single plane, horizontal wall and flat or slightly concave or convex oral surface; solitary Fan-shaped: both solitary and colonial Mushroom shaped; colonial Corallites separated by void space Creeping over some substrate, encrusting; colonial Nearly straight and of uniform diameter except in the apical region; solitary Like trochoid but with wider apical angle, about 70 degrees; solitary With still wider apical angle, about 120 degrees; broadly flattened conical in form; solitary The angle is about 40 degrees; solitary Flat base and highly convex oral surface; solitary Very slenderly conical, horn-shaped, the angle is only about 20 degrees; solitary Wedge-shaped; solitary Encrusting colony	916
Morphological	Distance between centres of corallites	no	The measured distance between the centres of two corallites	N/A	N/A	909
Morphological	Number of septal cycles	no	Number of cycles or orders in the mature corallite	N/A	N/A	647
Morphological	Height	no	The overall height of the specimen, usually a solitary coral	N/A	N/A	592
Morphological	Colony size	no	The maximum diameter of a colony	N/A	N/A	559
Morphological	Corallite width	no	Diameter of the corallite	N/A	N/A	428
Physiological	Zooxanthellate	yes	Whether the species is zooxanthellate (i.e., contains photosymbiotic zooxanthellae) or not Note: This is not directly observable and is inferred.	Zooxanthellate Azooxanthellate Apozooxanthellate	Contain zooxanthellae within their tissues Don't contain zooxanthellae within their tissues Sometimes contain zooxanthellae within their tissues	387
Reproductive	Budding type	yes	The position of new buds relative to the parent corallite wall Note: This is a morphological character that is directly observable	Intracalicular Extracalicular Both None	Occurring within the tentacle ring of the parent polyp Occurring outside the tentacle ring, with daughter corallites forming on the side of the parent corallite Both intra- and extracalicular No budding occurring	201

